# The invasive *Neisseria meningitidis* MenC CC103 from Brazil is characterized by an accessory gene repertoire

**DOI:** 10.1038/s41598-017-01671-x

**Published:** 2017-05-09

**Authors:** Michel Abanto Marin, Erica Fonseca, Fernando Encinas, Fernanda Freitas, Dhian Almeida Camargo, Roney Santos Coimbra, Ivano de Filippis, Ana Carolina Vicente

**Affiliations:** 1 Laboratório de Genética Molecular de Microrganismos, Instituto Oswaldo Cruz (IOC) - Fundação Oswaldo Cruz (FIOCRUZ), Av. Brasil, 4365, Rio de Janeiro, Brazil; 2 Fundação Ezequiel Dias (FUNED), Belo Horizonte, MG Brazil; 3 Neurogenômica, Centro de Pesquisas René Rachou (CPqRR), Fundação Oswaldo Cruz (FIOCRUZ), Belo Horizonte, MG Brazil; 4 Instituto Nacional de Controle de Qualidade em Saúde (INCQS), Fundação Oswaldo Cruz (FIOCRUZ), Rio de Janeiro, RJ Brazil

## Abstract

*Neisseria meningitidis* infections are a major issue for global health. The invasive MenC ST-103 clonal complex (CC103) has been the most prevalent in meningococcal outbreaks in Brazil, occurring also in several countries worldwide. Here we have analysed the population structure and accessory genome of MenC CC103 strains from a global perspective. An in-depth phylogenomic analysis revealed a lineage of *N. meningitidis* causing meningitis in Brazil and the United Kingdom. This lineage was also characterized as harbouring a particular accessory genome composed of CRISPR/Cas and restriction modification systems. This lineage was also characterized by a genomic island resembling an integrative and conjugative element. This island carried genes potentially associated with virulence and fitness. We propose this accessory gene repertoire could be contributing to the spatial-temporal persistence of the invasive MenC CC103 lineage.

## Introduction

*Neisseria meningitidis* is part of the commensal microbiota in the upper human respiratory tract; however, it can occasionally invade the bloodstream causing meningitis and/or septicemia, which are associated with significant morbidity and mortality worldwide. *N. meningitidis* are classified into 12 serogroups based on antigenic properties of polysaccharide capsules. Most invasive meningococci belong to the A, B, C, W, and Y serogroups^1^. The control of meningococcal disease has been approached worldwide with vaccination against the A, C, W, and Y serogroups and, more recently, against serogroup B strains^2^.

In the last decade, invasive meningococci from serogroup C (MenC) has increased in Brazil, with the ST-103 clonal complex (CC103) corresponding to over 74% of the disease causing strains characterized in 2014^3–5^. Due to this epidemiological scenario, the C serogroup conjugate vaccine was introduced in Brazil in 2010^6^. Despite this, however, cases and outbreaks caused by MenC CC103 have continued to be reported in Brazil^7^.

The worldwide epidemiology of MenC has been dynamic considering the spatial-temporal distribution of distinct clonal complexes. In Europe, this serogroup is the second most widespread, and MenC CC11 has been responsible for outbreaks in several countries, even after the introduction of the MenC vaccination^8^.

Although horizontal gene transfers between *Neisseria* species and other bacteria have a pivotal role in meningococcal evolution^9^, the *Neisseria* population structure is mainly represented by lineages, which may be determined by the expansion of strains belonging to a clonal complex and/or presence of restriction-modification (RMS) and Clustered Regularly Interspaced Short Palindromic Repeats (CRISPR) systems^10, 11^.

Comparisons of meningococcal genomes have allowed deep phylogenetic reconstructions and identification of accessory genomes that may be associated with invasive lineages; these accessory genomes represent a repertoire of genetic elements such as prophages, plasmids, and genomic islands^12, 13^. The Gonococcal genomic island (GGI) is a mobile element found in *N*. *gonorrhoeae* (80%) and in some *N. meningitidis* (17%) that is characterized by the presence of a particular Type 4 Secretion System (T4SS), which is supposed to play a role in *Neisseria* virulence and pathogenesis^14–17^.

The aim of this study was to determine the population structure and the accessory genome of invasive MenC CC103 genomes from Brazil under a global epidemiological perspective. We found that Brazilian MenC CC103 strains belong to a lineage characterized by CRISPR spacer sequences and a set of restriction modification systems. This lineage also harbour a genomic island resembling an integrative and conjugative-like element carrying genes predicted to code for virulence- and fitness-associated functions.

## Results and Discussion

### Genome statistics and phylogenomic analysis

The genome sequences of 24 invasive MenC strains from Brazil, belonging to the ST-103 clonal complex (CC103), were determined in this study. The genomes have been assembled into between 70 to 183 contigs, totaling, on average, ~2.17 Mb with a G + C content of ~51.5%; they are predicted to encode ~2,200 genes with 4 rRNA operons and 51 tRNA genes. A detailed statistics of these genomes is summarized in Table S1. The genomes were classified by multilocus sequence typing (MLST) as belonging to the CC103 (Table 1). Studies have shown that MenC of CC103 has caused outbreaks in Brazil in the last few years^18^.

**Table 1:** MenC strains from Brazil sequenced in this study.

Strain	State/Country^1^	Year	porA	PorA VR1	PorA VR2^2^	porB	fetA	nadA^3^	nhba	fHbp	ST	Clonal Complex
Nm56	MG/Brazil	2011	28 (22)	22	2-48	2-23	F3-9	96	24	25 (25)	3780	ST-103
Nm287	MG/Brazil	2011	28 (22)	22	2-48	2-23	F5-7	85	24	25 (25)	3780	ST-103
Nm288	MG/Brazil	2011	28 (22)	22	2-48	2-23	F5-7	161	24	25 (25)	3780	ST-103
Nm292	MG/Brazil	2011	28 (22)	22	2-48	2-23	F5-7	161	24	25 (25)	3780	ST-103
Nm612	MG/Brazil	2011	28 (22)	22	2-48	2-23	F5-7	85	24	25 (25)	3780	ST-103
Nm638	MG/Brazil	2011	28 (22)	22	2-48	2-23	F5-7	85	24	25 (25)	3780	ST-103
P3478	BA/Brazil	2009	10-1	5-1	10-1	2-23	F3-9	85	24	25 (25)	8436	ST-103
NmCRJ2	RJ/Brazil	2012	28	22	2-48	2-23	F3-9	96	24	25 (25)	3779	ST-103
Nm94	MG/Brazil	2011	28 (22)	22	2-48	2-23	F3-9	96	24	446 (377)	3780	ST-103
Nm322	MG/Brazil	2011	28 (22)	22	2-48	2-23	F3-9	161	24	446 (377)	3780	ST-103
P3966	PE/Brazil	2011	28 (22)	22	2-48	2-253	F3-9	85	24	25 (25)	3780	ST-103
P3978	PE/Brazil	2011	28 (22)	22	2-48	2-23	F3-9	85	24	25 (25)	3780	ST-103
P4005	PE/Brazil	2012	28	22	2-48	2-23*	F3-9	96	24	25 (25)	3780	ST-103
P4144	PE/Brazil	2012	28	22	2-48	2-23	F3-9	85	24	25 (25)	3780	ST-103
P3558	PE/Brazil	2010	28	22	2-48	2-23	F3-9	85	24	25 (25)	3780	ST-103
P3965	PE/Brazil	2011	28	22	2-48	2-23	F3-9	96	24	25 (25)	ND	-
P4077	RJ/Brazil	2012	28	22	2-48	2-23	F3-9	85	24	25 (25)	5122	ST-103
P4431	NI/Brazil	2014	28	22	2-48	2-23	F3-9	161	24	25 (25)	3780	ST-103
P4615	NI/Brazil	2015	714	21	16-36	3-106	F5-8	85	24	490 (420)	3327	ST-865
P4534	NI/Brazil	2015	28	22	2-48	2-23	-	96	24	25 (25)	3779	ST-103
P4480	NI/Brazil	2015	28	22	2-48	2-23	F3-9	85	24	25 (25)	3780	ST-103
P4950	NI/Brazil	2015	28	22	2-48	2-23	-	85	24	25 (25)	3779	ST-103
P4995	NI/Brazil	2015	28	22	2-48	2-23	F3-9	85	24	25 (-)	3780	ST-103
P4464	NI/Brazil	2014	28	22	2-48	2-23	F3-9	85	24	789 (-)	8730	-

The antigen-encoding gene profiles, sequence type and clonal complex designations were defined based on Neisseria pubMLST database (http://pubmlst.org/neisseria/). ^1^MG, Minas Gerais; BA, Bahia; RJ, Rio de Janeiro; PE, Pernambuco; NI, not informed. ^2^The 2-48 profile was defined by the closest match locus ^3^ *nadA* locus interrupted by insertion sequence (IS) element; the profile was defined by the closest match locus; -, not defined by PubMLST search.

**Figure 1:**
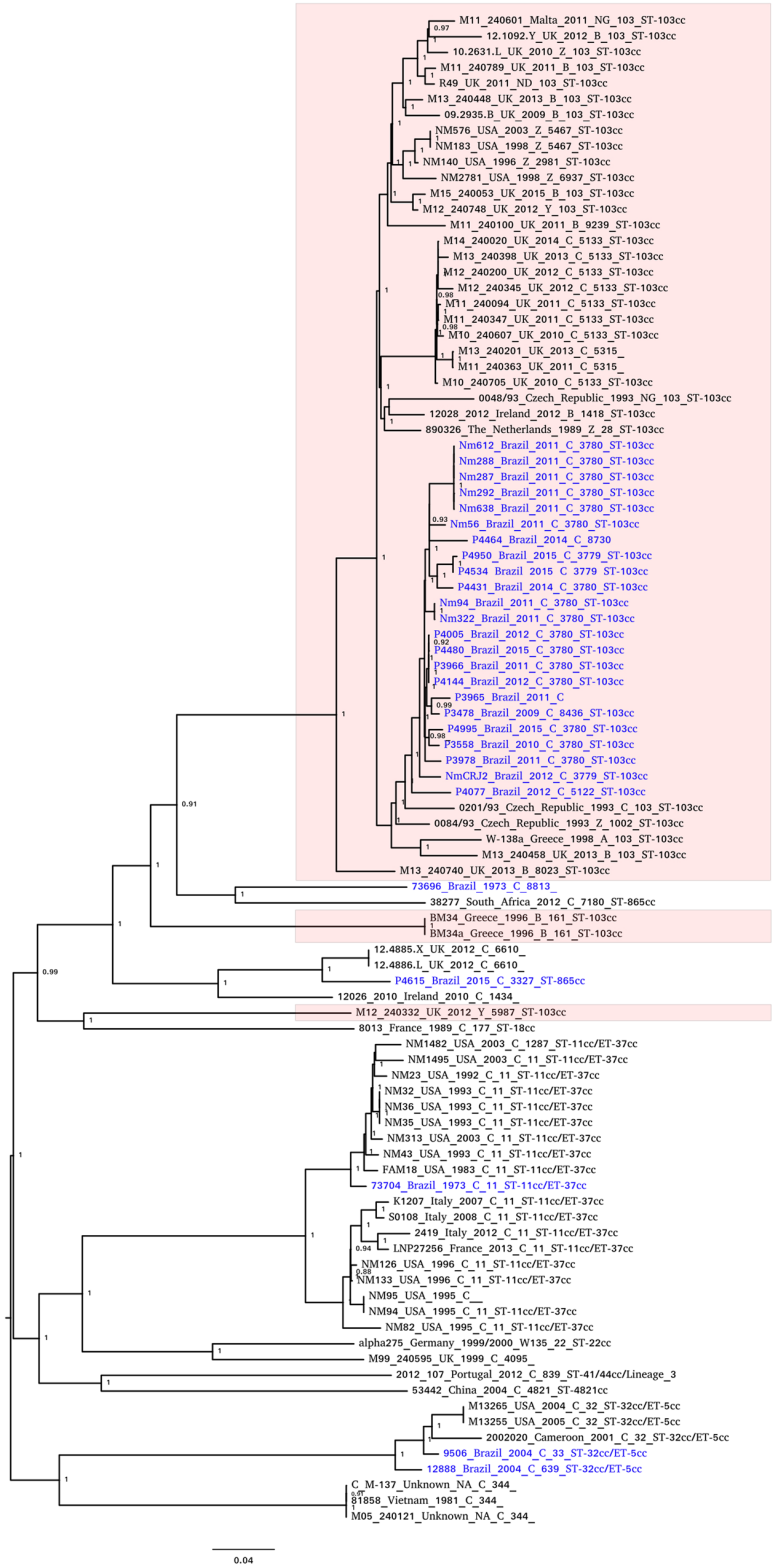
Genome phylogeny overview of *N. meningitidis* CC103 complex and close relatives. The phylogeny was determined considering the core genome alignment and maximum likelihood reconstruction phylogeny by using Parsnp program implemented in Harvest suite^55^. Highlighted boxes indicate the genomes assigned as CC103 complex. Brazilian MenC strains are depicted in blue.

An initial phylogenomic analysis was performed with 645 MenC genomes retrieved from the Bacterial Isolate Genome Sequence Database (BIGSdb) (see Fig. S1). To gain a better understanding of the evolution and population structure of CC103, 96 representative genomes were selected from this preliminary phylogeny for a more detailed analysis. Of these 96 MenC strain genomes, 24 were from Brazil (Fig. 1, Table S2). The great majority of CC103 genomes analysed belong to a cluster of invasive *N. meningitidis* strains from serogroups A (n = 1), Y (n = 1), B (n = 9), C (n = 36), Z (n = 6), and non-groupable (n = 5) strains identified in the last three decades in Brazil and European countries. The presence of different serogroups in this cluster indicated the occurrence of capsular switching events. All MenC CC103 Brazilian strains were placed in a subcluster, which allowed them to be classified to a defined lineage (Fig. 1).

### The accessory genome of MenC CC103 - Prophage and CRISPR/Cas identification

**Figure 2:**
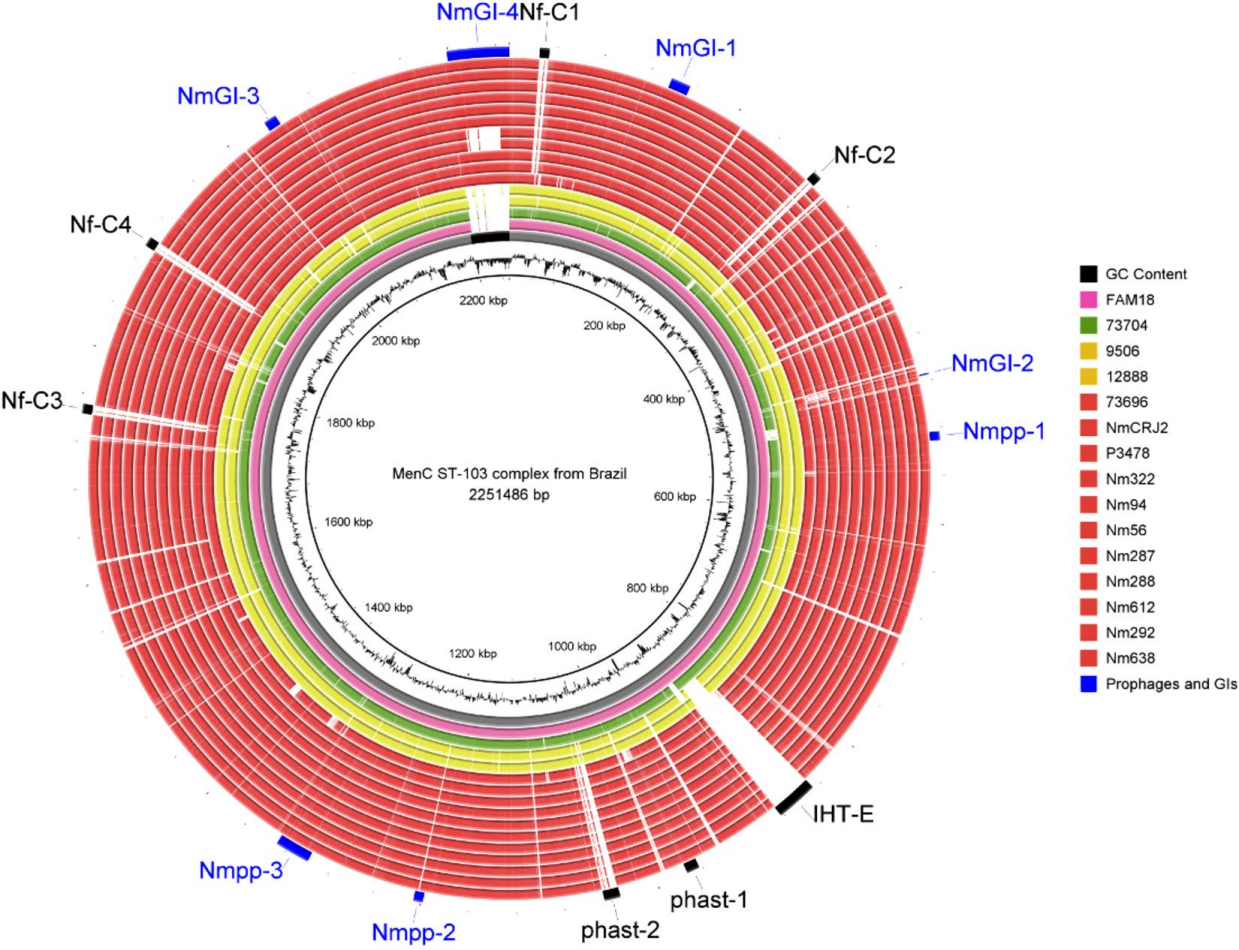
Circular representation of the genomes from *N*. *meningitidis* serogroup C (MenC) strains isolated from Brazil. The draft genomes were aligned against FAM18 reference genome. The inner circle (histogram) shows G + C content variations throughout the genome. The gray circle represents the compared chromosomal regions and the black region placed in the terminus region ($\sim 2$,200 bp) represents regions not allocated as part of the chromosomal genome. Each genome is represented by a colored circle. The red circles represent Brazilian MenC genomes from CC103 clonal complex harbouring the ICE*Nm*CC103. The variable regions such as prophages and genomic islands, including the NmGI-4 (ICE*Nm*CC103 element), are highlighted.

Comparative genomic analyses of the MenC CC103 from Brazil and the FAM18 reference genomes revealed the presence of four genomic islands (NmGI-1 to 4) and ten prophages (Nf-C1, Nf-C2, IHT-E, Nf-C3, Nf-C4, Nmpp-1, phast-1, phast-2, Nmpp-2, and Nmpp3) (Fig. 2). All four genomic islands and 4/10 prophage sequences (Nmpp-1, phast-1, Nmpp-2, and Nmpp3) were found in the MenC CC103 genomes from Brazil while all these genomic regions were present in FAM18, except NmGI-4. Although the prophages Nf-C1, Nf-C2, IHT-E, Nf-C3, Nf-C4 and phast-2 identified in FAM18 reference genome had already been associated with different invasive serogroups^19–21^, none of them were present in the Brazilian invasive MenC CC103 lineage (Fig. 2, Fig. S3), corroborating the polygenic character of meningococcal virulence^13^.

Bacteria possess CRISPR/Cas systems that can restrict horizontal gene transfer, such as phage insertion by transduction^22^. We identified a CRISPR/Cas system in the MenC CC103 from Brazil (Table S1) that was absent in the FAM18 genome. This identified system consisted of Cas proteins and arrays of a 36 nucleotide identical direct repeat (ATTGTAGCACTGCGAAATGAGAATGGGAGCTACAAC), separated by variable spacer regions. The number of direct repeats was seen to be variable among these genomes (vary from 12 to 28) as was the sequence composition of the spacer regions, which ranged from 28 to 42 bp in length. Interestingly, some of these spacer sequences corresponded to 5/6 prophages identified in FAM18 that were missing in the Brazilian MenC CC103 lineage (Nf-C1, Nf-C2, Nf-C3, phast-2 and Nf-C4). This finding suggests that this CRISPR/Cas system could limit the acquisition of these phages by the Brazilian MenC CC103 lineage (Fig. 2), and could be contributing to its population structure delineation.

### General features of the NmGI-4 genomic island

**Figure 3:**
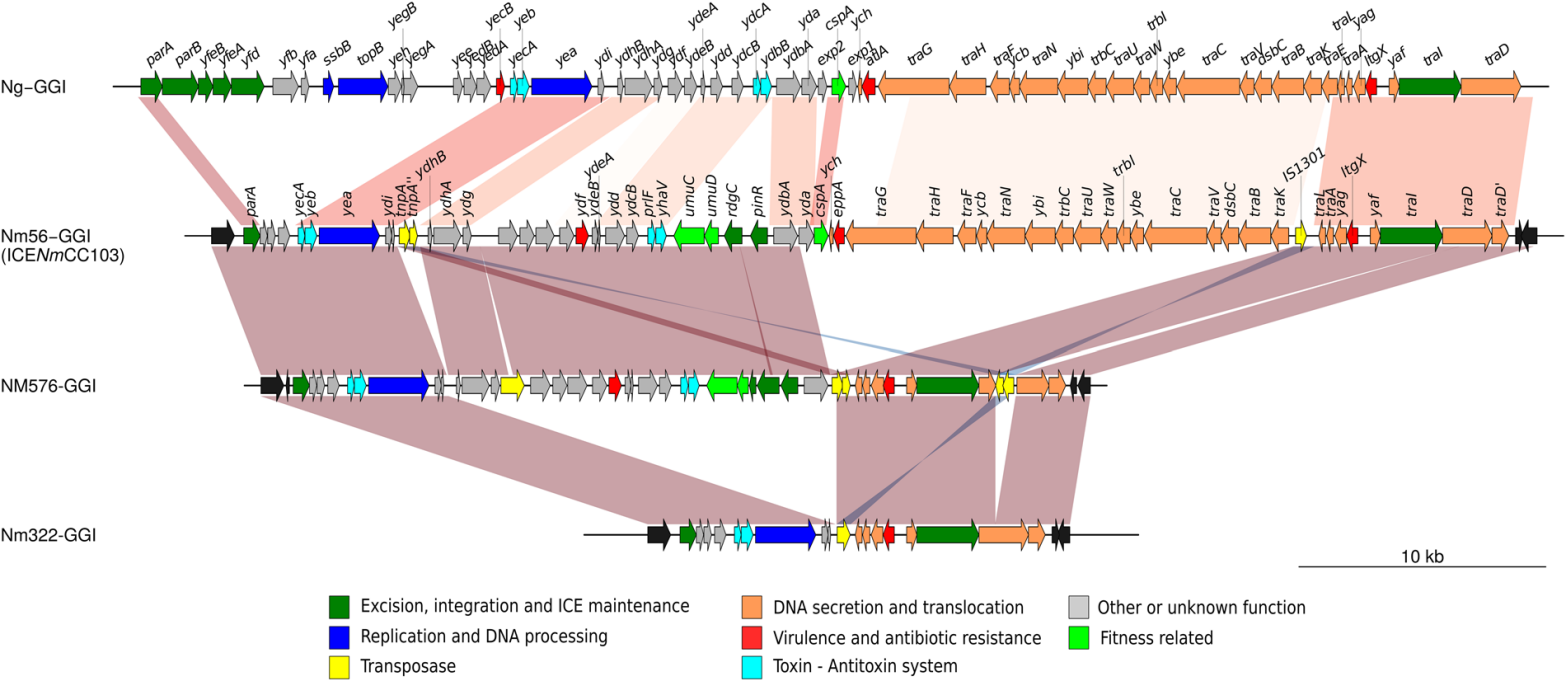
Genetic organization of the GGI from *N*. *gonorrhoeae* (Ng) compared with the *N. meningitidis* GGI-like elements characterized in this study. Schematic comparison of the GGI versions of Ng, Nm56 (ICE*Nm*CC103), Nm576 and Nm322 genomes. Genes are presented as arrows, with the arrowhead indicating the direction of the transcription. Black arrows indicate the chromosomal genes flanking the GGI-like elements, which are represented by colored arrows. Areas between the GGIs shaded as horizontal blocks indicate similar regions based on sequence identity.

The NmGI-1-3 was shared by all genomes included in this comparative analysis. Conversely, the NmGI-4 was specific for MenC CC103 lineage from Brazil (Fig. 2). This genomic island is ~52.3 kb in length, has a G+C content of 44% and it was flanked by identical 23 bp direct repeat sequences. It was observed that the NmGI-4 had several features in common with the Gonococcal Genomic Islands (GGIs), which are mobile elements characterized by the presence of a particular T4SS^23^. For example, the NmGI-4 shared synteny and gene content with GGIs and they were inserted in the same chromosomal context (*dif* site) (Fig. 3, Fig. S2).

Different GGI configurations have already been identified in *N. meningitidis* ^12, 23, 24^ and, in fact, the comparative genomic analysis performed here revealed the presence of different GGI-like versions among the MenC CC103 strains. The NmGI-4 (complete version recovered from Nm56 strain) shared 97% coverage and 99% nucleotide identity with the GGI-like T4SS characterized in *N. meningitidis* serogroup W alpha-275 strain^25^. Two GGI variants were also identified (recovered from Nm576 and Nm322), and they differ from the NmGI-4 and from the types previously described in *N. meningitidis* ^25^ by the presence of large deletions (Fig. 3).

### The unique genetic repertoire of NmGI-4 genomic island

NmGI-4 harboured a set of genes including some that could have an adaptive role in strains carrying it such as *umuC*, *umuD*, *rdgC*, and *pinR* (Fig. 3).

Meningococci residing in the nasopharynx are exposed to oxidative stress that can cause DNA damage, reflecting the need of DNA repair mechanisms. However, this species lacks the SOS response system such as the UmuDC-dependent mutagenic repair^26, 27^. Interestingly, NmGI-4 carried the *umuDC* system, which represents an additional DNA repair pathway/mechanism and could be associated with fitness innovation^28^. The *rdgC* gene found in NmGI-4 is a homolog of the DNA recombination-dependent growth factor C (RdgC), whose deduced protein shared 70–90% amino acid identity with other RdgC alleles present in the chromosome of several *Neisseria* species. RdgC modulates RecA activity, being involved in several cell functions such as pilin antigenic variation, natural DNA transformation, and SOS DNA repair induction^29–31^. The presence of *rdgC* together with the *umuDC* system in the context of NmGI-4 suggests that the former may control the SOS response evoked by UmuDC, contributing to the adaptive fitness of MenC CC103. Finally, the *pinR* gene encodes a site-specific resolvase from the Serine Recombinase Superfamily, which is involved in excision/insertion of entire genomic islands. Therefore, contrasting with the GGIs so far described in *N*. *gonorrhoeae*, which lack any site-specific recombinase gene and depend on an *in trans* activity of the XerCD recombinase to be excised/integrated^32^, the presence of *pinR* in the NmGI-4 potentially provide the ability of self-excision/insertion through *in cis* recombination events.

Integrative and conjugative elements (ICEs) are defined by the presence of genes involved in integration/excision, conjugation and replication functions^33^. Therefore, in spite of the similarities between NmGI-4 and GGIs (Fig. 3), the former contained a serine recombinase *pinR* gene (integration/excision module), the entire set of T4SS genes (conjugation/secretion module), the *parA* gene and the transfer origin *oriT* (replication module) that, altogether, would characterize the NmGI-4 as an ICE (Fig. 3)^33^. Based on these features, we named this NmGI-4 version (complete version found in Nm56 strain) as ICE*Nm*CC103 (relative to the ST-103 clonal complex).

Together with the *umuDC* genes, potentially involved in adaptive traits, several genes that could contribute to the success of a pathogen in colonizing and infecting its host were found in ICE*Nm*CC103, such as the *eppA* gene and toxin-antitoxin (TA) systems. The *eppA* encodes a zinc metallopeptidase/endopeptidase from the M23 family. Proteins from this enzyme class may act in a variety of processes, including pilus DNA secretion process, cleavage of the extracellular matrix fibronectin from the host’s tissue and pilus biogenesis^34–36^.

TA modules comprise toxin proteins, which disrupt the cell’s own molecular processes, and cognate antitoxins that block this poisonous activity. These systems can be found in chromosomes and within mobile genetic elements including plasmids, prophages, transposons or superintegrons^37^. TA systems are activated in response to environmental stress, and confer advantageous bacterial behaviours such as biofilm formation, phase variation, virulence regulation, bacterial persistence, genetic competence and plasmid maintenance during cell division, which, in turn, may contribute to the dissemination of antibiotic resistance and virulence determinants harboured by such mobile genetic elements^37, 38^. TA systems are part of *N. meningitidis* genetic background and vary among lineages^39^. Using TA-Finder software (http://202.120.12.133/TAfinder/), we verified that MenC CC103 and FAM18 share TA systems from the *RelE*, *Xre*, *MazF*, and *AbrB* families while the PIN-PHD system was restricted to the FAM18 genome. Moreover, two additional TA systems, *prlF-yhaV* ^40^ and *yecA-yeb*, were identified embedded in ICE*Nm*CC103 (Table S3). Therefore, the presence of two other TA systems in ICE*Nm*CC103 suggests that, besides their contribution to ICE maintenance, they can provide an adaptive increment to the CC103 lineage.

### Genomic epidemiology of ICE*Nm*CC103

**Figure 4:**
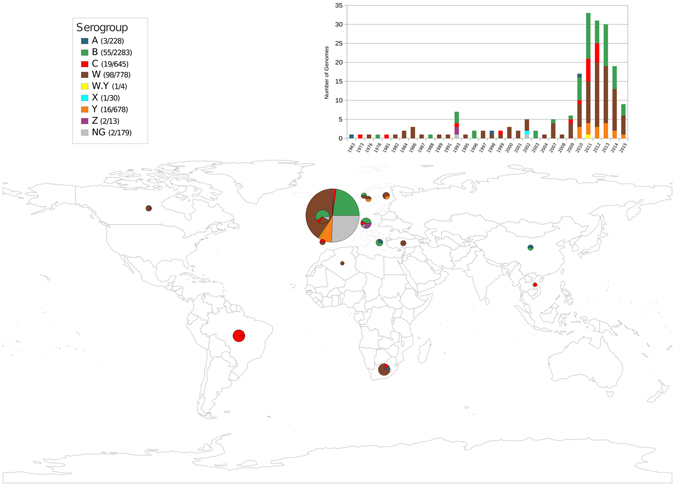
ICE*Nm*CC103 geographical distribution and its abundance among *N. meningitidis* genomes from distinct serogroups. This figure has been created in R^51^ using the Rworldmap package^52^. The legend on the top left side shows the number of genomes positive for ICE*Nm*CC103 element relative to the total number of genomes of each serogroup. On the top right the prevalence of this element in genomes is shown for different serogroups (different colored bar sections) and year of isolation.

We performed an analysis focusing on the prevalence and worldwide distribution of ICE*Nm*CC103 using this element as a query to search the *Neisseria* sequence repository at the PubMLST database. A total of 247 meningococci genomes harbouring the ICE*Nm*CC103 were identified (Fig. 4). The highest ICE prevalence was in serogroup W (n = 98), followed by B (n = 55), C (n = 19), and Y (n = 16) with a bias to genomes from European countries, particularly the United Kingdom (Fig. 4). A phylogenomic analysis using 96 genomes from CC103 and closely related clonal complexes revealed that most of them belong to a monophyletic cluster and carried ICE*Nm*CC103 (Fig. 4), showing a global association of ICE*Nm*CC103 with CC103 strains. This cluster was mainly composed of genomes from the United Kingdom and Brazil recovered from meningitis cases between 1973 and 2015. Interestingly, the presence of other serogroups (A, B, W and Z), besides MenC, in this CC103 cluster indicates the occurrence of capsular switching events involving strains from this clonal complex.

**Figure 5:**
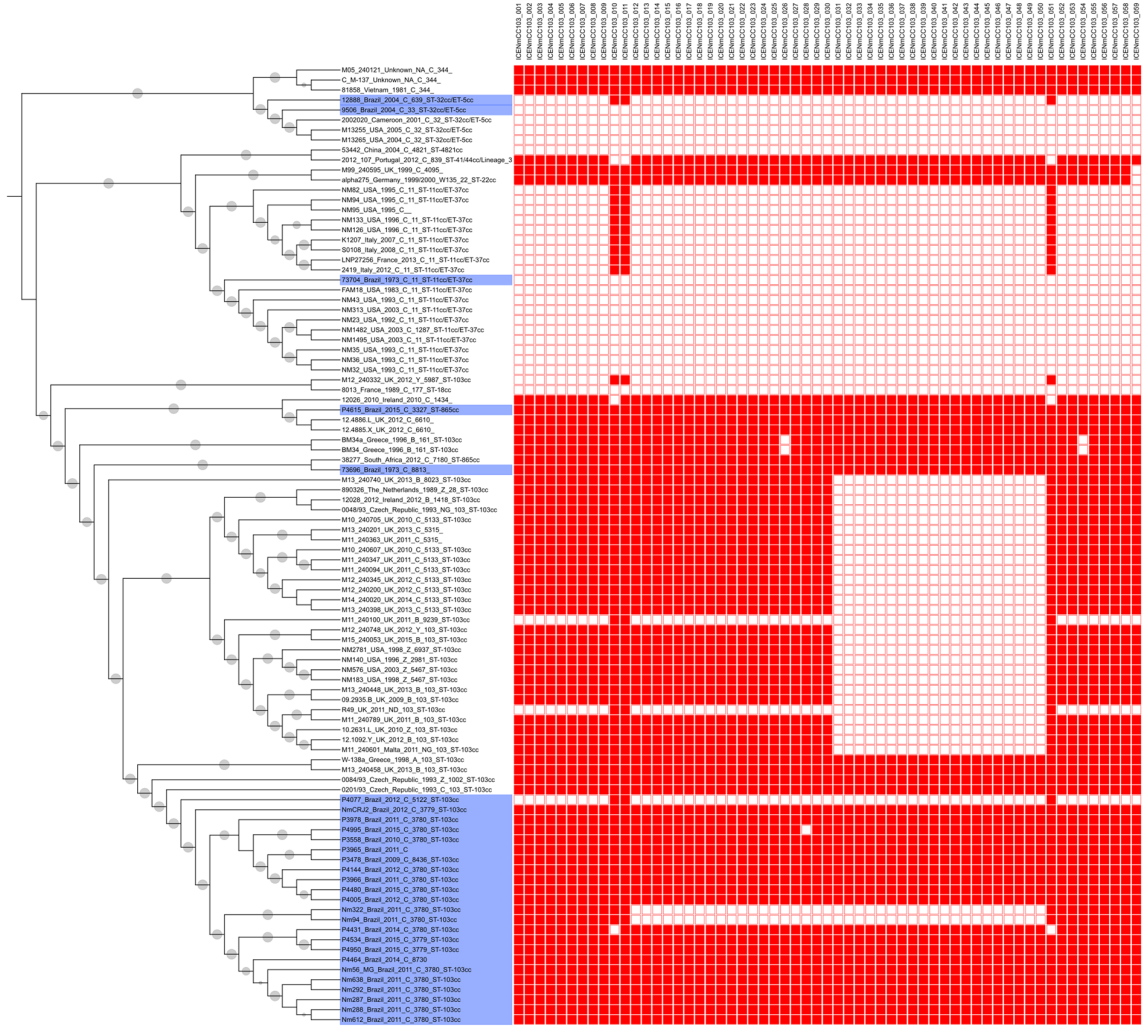
Phylogeny of *N. meningitidis* from CC103 and close relatives associated with a heatmap of the ICE*Nm*CC103 gene content. The genes listed in the top correspond to ICE*Nm*CC103 genes. The gene content is represented as presence (red) or absence (white). Most of CC103 genomes, including genomes from Brazil, belong to a monophyletic cluster and carried the ICE*Nm*CC103 element. All genomes from Brazil are shaded in blue.

Two other islands related to ICE*Nm*CC103 were also identified: one characterized by a 37 kb deletion, was found in the Brazilian Nm94 and Nm322 genomes (Fig. 3), and the other, characterized by a 20 kb deletion, was found in 26 genomes mainly derived from the United Kingdom strains (Fig. 5).

### Restriction modification systems (RMS) in MenC CC103

**Figure 6:**
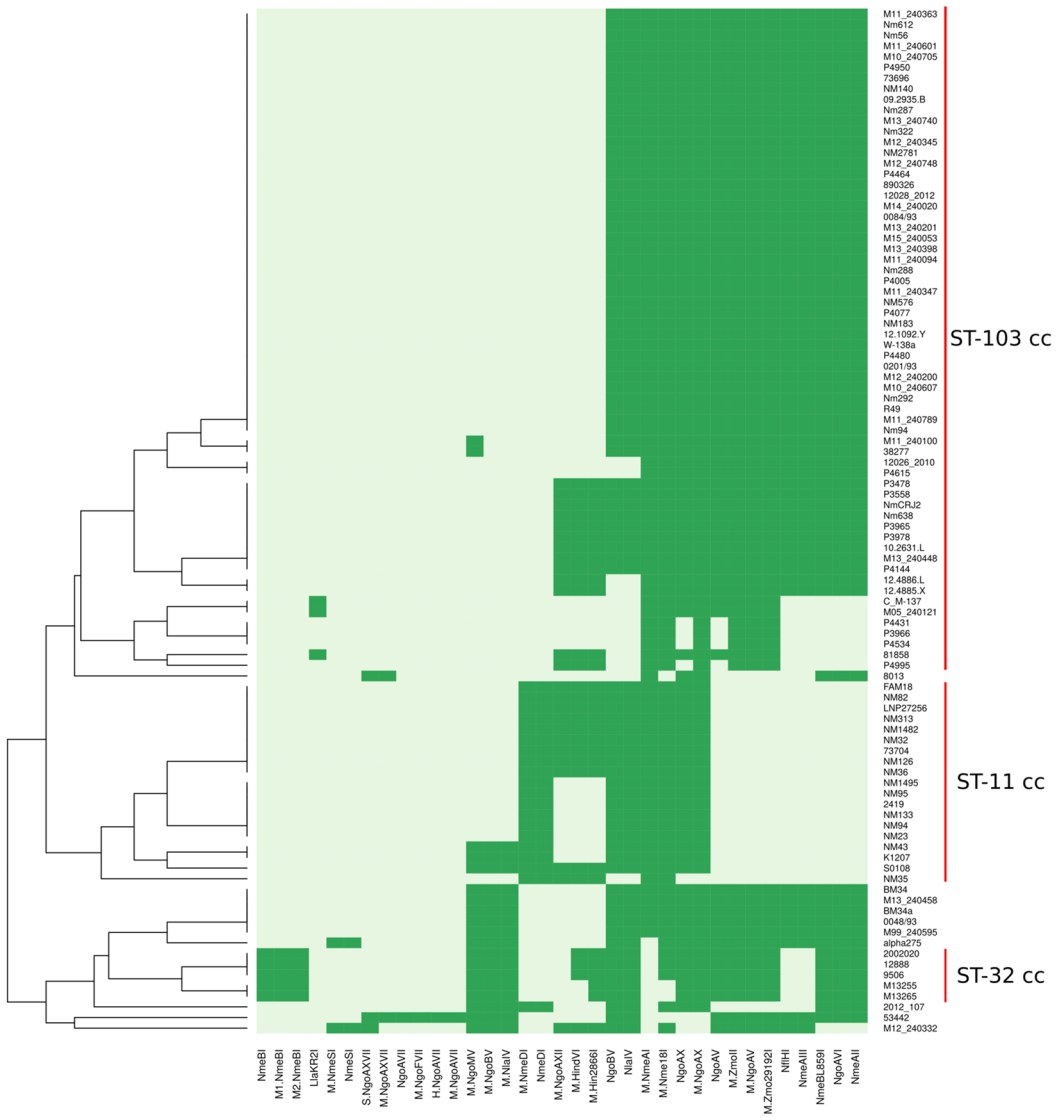
Heatmap showing the presence/absence of RMSs in *N. meningitidis* genomes. Each row in the matrix represents one genome and each column represents one of 35 RMSs identified. The names of RMSs correspond to the reference names used in REBASE database. A dark green square indicates the presence of an RMS in the genome, while light green indicates absence. The dendrogram on the left was generated by applying a hierarchical clustering algorithm where the genomes are grouped according to their RMS content.

In order to determine if the CC103 lineage is characterized by a specific combination of RMS, we applied a hierarchical clustering algorithm using the presence/absence of genes from the REBASE database. In general, the clusters defined by RMS patterns corresponded to those revealed by the phylogenomic analysis (Fig. 6). The CC103 lineage was characterized by a specific RMS pattern: M.NmeAI, M.Nme18I, NgoAX, M.NgoAX, NgoAV, M.Zmoll, M.NgoAV, M.Zmo29192I, NflHI, NmeAIII, NmeBL869I, NgoAVI, and NmeAII. A previous study demonstrated that the population structure of the CC41/44, PC32/69, and PC8/11 phyloclades is shaped by specific RMS patterns, and the authors hypothesized that this could be a global phenomenon in populations of *N. meningitidis* ^10^. Our results showed that a lineage from another clonal complex (CC103) is also delineated by these systems. Therefore, our findings are adding evidences to the role of RMS in maintaining the population structure of *N*. *meninigitidis*.

In the present study, we performed an in-depth phylogenetic analysis using the core genome information of *N. meningitidis*. This analysis revealed that *N. meningitidis* strains from CC103 that were characterized by an accessory genome belong to several different serogroups (Fig. S4). This study contributes to the understanding of *N. meningitidis* clonal complexes evolution based on their pangenomes. Therefore, population studies on meningococci should include analyses of the core as well as the accessory genomes, which drive the fast evolution of lineages and also contributes to the emergence of new phenotypes.

## Methods

### Strain collection, bacterial culture, and DNA isolation

The 24 invasive *N. meningitidis* strains used in this study (Table 1) were recovered from several meningitis cases treated in hospitals in four Brazilian regions: Bahia (2009), Pernambuco (2010–2012), Minas Gerais (2011), and Rio de Janeiro (2012). The isolates were grown on 5% sheep blood agar. Each subcultivation was started from a single colony, and several colonies were used for genomic DNA extraction with the ChargeSwitch gDNA Mini Bacteria Kit (Invitrogen), according manufacturer’s instructions. Approvals from the institutional review board of the hospitals and the Research Ethics committee of the Meningitis Advisory Committee of the State Department of Health from Rio de Janeiro, Brazilian Ministry of Health in Bahia, and Ezequiel Dias Foundation in Minas Gerais were obtained to conducting the study. All methods were performed in accordance with the required guidelines and regulations.

### Genome sequencing, assembly annotation, and analysis

Genomic DNA was sequenced on an Illumina HiSeq 2500 sequencer using Nextera XT paired-end run with a 500-bp insert library at the High-Throughput Sequencing Platform of the Oswaldo Cruz Foundation (Fiocruz, Rio de Janeiro, Brazil). Following quality assessment of the reads with FASTQC, assemblies were generated using the SPAdes 3.5 assembler^41^. Improved assemblies were produced with the PAGIT pipeline using default settings^42^.

### Mobile elements

We identified putative genomic islands and prophage regions by using the IslandViewer (http://www.pathogenomics.sfu.ca/islandviewer/)^43^ and PHAST (http://phast.wishartlab.com/)^44^ software, respectively. Genome comparisons were performed in the BRIG program (http://brig.sourceforge.net/)^45^.

Putative ICEs were identified by the presence of a modular organization of a typical ICE^33^, such as the presence of *oriT*, DNA recombinase, relaxase, and T4SS. Contigs from Nm56 genome that harboured an ICE-like region were delimited, concatenated, and compared with other assembled genomes to reconstruct a putative ICE. A putative ordered ICE-region was used as a template for mapping the reads using Bowtie2 (http://bowtie-bio.sourceforge.net/bowtie2)^46^. Paired-end reads, corresponding to the ICE target, were recovered with SAMtools (http://samtools.sourceforge.net)^47^ and finally assembled with SPAdes 3.5 using default settings. The final contig was confirmed to be complete by PCR using custom primers; PCR amplification products were sequenced by Sanger sequencing. The ICE-like region of Nm56 was annotated using Prokka 1.8^48^ with subsequent manual curation in Artemis (www.sanger.ac.uk/). The final representation and comparison with other ICE-like elements were performed using genoplotR^49^. We performed Blast searches using the ICE-like element of Nm56 as a query against all BIGSdb genomes. The element was considered present when hits with sequence identities above 70% and more than 10,000 identical residues were obtained. The LS-BSR pipeline^50^ and the R Package^51^ were used to detect the presence of ICE-like elements in MenC genomes (Fig. S1), as well for genomic comparison with the GGI from *N*. *gonorrhoeae* (Fig. S2). The geographical visualization of *N. meningitidis* genomes carrying ICE-like region was done with rworldmap^52^.

### Phylogenomic analysis

All 5,531 genomes of *N*. *meningitidis* and the metadata used in this study were retrivied from BIGSdb database^53^ (www.pubmlst.org/neisseria) in September 2015. The *csc* gene, which encodes a particular poly sialyltransferase that defines the serogroup C, was used as a query in the Blastn search against BIGSdb to select all MenC genomes. A preliminary genome phylogeny with 645 MenC genomes was performed by feature frequency profiles - FFPs (http://ffp-phylogeny.sourceforge.net/)^54^ (Fig. S1). From the resulting phylogeny, 96 representative MenC genomes and other *N. meningitidis* CC103 close relatives (considering the presence in GenBank and their position at the phylogenetic tree) were selected for a more datailed analysis (Table S2). Core genome SNPs were identified in these genomes and used for the construction of a phylogenetic tree with the Parsnp program from the Harvest suite^55^ with the “-p 16 -x -c” parameters. The Gegenees software^56^ was used for the comparative analysis of gene contents among genomes. The software performs a pairwise comparison of genome fragments creating a distance matrix based on shared fragments, which was exported as a Nexus file; the split tree gene content was produced using SplitsTree4 through the GeneContentDistance method^57^.

### Identification of DNA methyltransferase genes/RMS analysis

All nucleotide sequences of RMSs (DNA methylase and DNA endonuclease) identified in *N. meningitidis* were obtained from REBASE (rebase.neb.com). Total sequence redundancy (100% identity) were removed using the CD-HIT-EST program^58^. An RMS presence/absence matrix, considering the 35 non-redundant RMSs sequences, was constructed with the LS-BSR pipeline using default settings^50^. The resulting presence/absence binary table was converted into a distance matrix and clustered by means of a complete linkage hierarchical cluster analysis using heatmap.2 function from the gplots R package^51^.

### CRISPR identification and analyses

Putative CRISPR arrays were identified in the Brazilian MenC CC103 using PILER-CR v1.06 with default parameters^59^. Spacer target sequences were clustered with CD-HIT v4.6 using a 90% sequence identity cutoff (*parameter*: *cd* − *hit* − *est* *−* *i*[*spacer sequences*] − *r*0 − *c*0.9 − *d*100 − [*result*]); the non-redundant spacer sequences were subsequently compared with prophage regions found in the FAM18 genome by similarity comparisons.

## Electronic supplementary material

Supplemental information
