# Supplementary material for: The invasive Neisseria meningitidis MenC CC103 from Brazil is characterized by an accessory gene repertoire
Source: Sci Rep. 2017 May 9;7:1617. doi: 10.1038/s41598-017-01671-x (PMC5431661; doi:10.1038/s41598-017-01671-x)
Supplement: Supplementary file 1 — Supplemental information [file 41598_2017_1671_MOESM1_ESM.pdf]

The invasive *Neisseria meningitidis* MenC CC103 from Brazil is characterized by an accessory gene repertoire

Michel A. Marin[1,*], Erica L Fonseca[1], Fernando Encinas[1], Fernanda Freitas[1], Dhian Almeida Camargo[2], Roney Santos Coimbra[3], Ivano de Filippis[4], Ana Carolina P. Vicente[1]

[1] Laboratório de Genética Molecular de Microrganismos, Instituto Oswaldo Cruz (IOC) - Fundação Oswaldo Cruz (FIOCRUZ), Av. Brasil, 4365, Rio de Janeiro, CEP 21045-900, Brazil.

[2] Fundação Ezequiel Dias (FUNED), Belo Horizonte, MG, Brazil

[3]Neurogenomica, Centro de Pesquisas Rene Rachou, FIOCRUZ. Av. Augusto de Lima, 1715, Barro Preto, Belo Horizonte, MG, Brazil, 30190-002

[4]Instituto Nacional de Controle de Qualidade em Saúde - INCQS, FIOCRUZ, Rio de Janeiro, RJ, Brazil

* To whom correspondence should be addressed. Tel: +55-21-38658168; Fax: +55-21-22604282; E-mail: mfabanto@gmail.com

Email addresses:

MAM: mfabanto@gmail.com

ELF: ericafon@ioc.fiocruz.br

FE: ferenpo@ioc.fiocruz.br

FF: freitasf@ioc.fiocruz.br

DC: dhianrenato@gmail.com

RSC: roney.s.coimbra@cpqrr.fiocruz.br

IDF: ivano.defilippis@incqs.fiocruz.br

ACPV: anapaulo@ioc.fiocruz.br

Present Address: Laboratório de Genética Molecular de Microrganismos, Instituto Oswaldo Cruz (IOC) - Fundação Oswaldo Cruz (FIOCRUZ), Av. Brasil, 4365, Rio de Janeiro, CEP 21045-900, Brazil

Supplementary information

Supplementary figure legends

Figure S1. Phylogenomic tree performed with feature frequency profiles (FFPs) considering 645 MenC genomes. The gene content of NmGI-4 (ICE*Nm*CC103) associated with the tree is shown as presence (blue) or absence (white). Only a subset of MenC clades harbors this element.

Figure S2. Heatmap showing ICE-like gene content between 1585 genomes (1338 *N. gonorrhoeae* and 247 *N. meingitidis*) had positive hits with ICE*Nm*CC103. Gene content comparison between them shows two major profiles, corresponding to Ng and Nm, respectively.

Figure S3. Prophages in *N. meningitidis* genomes. Presence and absence of the Nf-C1, Nf-C2, IHT-E, Nf-C3, and Nf-C4 prophages (right) across the phylogeny of the Nm genomes (left). Presence and absence of the prophages are indicated by filling and empty blue boxes, respectively. Genomes belong to clonal complex CC103 from Brazil were characterized by the absence of these prophages.

Figure S4. Network phylogeny of MenC genomes based on gene content. MenC from distinct clonal complexes were distinguished in different clusters, including the ICE*Nm*CC103-positive CC103 genomes. This result suggests that *N. meningitidis* from CC103 is a lineage formed by strains sharing the same accessory gene repertoire. Genomes were compared according to a pairwise comparison of genome fragments by using the Gegenees and SplitsTree4 softwares.

Table S1. Genomic traits based on assembly and gene annotation of *N. meningitidis* genomes sequenced in this study.

Strain	BioSample	BioProject	Accession	Length (bp)	Coverage (x)	Scaffolds	Genes (total)	CDS (coding)	Genes (RNA)	rRNA	tRNA	ncRNA	Pseudo Genes	CRISPR Arrays
P4615	SAMN05413707	PRJNA329301	MBLW00000000	2.176.725	688	135	2279	2031	61	3	54	4	187	1
P3966	SAMN05413708	PRJNA329301	MBLX00000000	2.214.913	487	116	2303	2071	61	4	53	4	171	2
P3978	SAMN05413724	PRJNA329301	MBLY00000000	2.206.946	343	78	2292	2086	59	2	53	4	147	1
P4077	SAMN05413737	PRJNA329301	MBLZ00000000	2.207.225	728	183	2315	2050	68	9	55	4	197	1
P4431	SAMN05413738	PRJNA329301	MBMA00000000	2.203.425	686	87	2319	2069	66	8	54	4	184	1
P4464	SAMN05413763	PRJNA329301	MBMB00000000	2.217.808	786	131	2343	2066	60	3	53	4	217	2
P4480	SAMN05413765	PRJNA329301	MBMC00000000	2.199.042	29	70	2305	2088	61	4	53	4	156	2
P4534	SAMN05413836	PRJNA329301	MBMD00000000	2.231.661	495	101	2353	2101	67	12	51	4	185	1
P4950	SAMN05414884	PRJNA329301	MBME00000000	2.225.395	120	83	2332	2106	64	8	52	4	162	2
P4995	SAMN05414886	PRJNA329301	MBMF00000000	2.211.148	697	107	2313	2086	63	5	54	4	164	2
P4005	SAMN05415277	PRJNA329301	MBMG00000000	2.194.619	561	134	2293	2056	60	3	53	4	177	2
P3558	SAMN05410591	PRJNA329301	MBKM00000000	2.181.182	329	112	2048	2048	59	3	52	4	167	1
P3965	SAMN05410612	PRJNA329301	MBKN00000000	2.202.968	427	159	2331	2062	61	4	53	4	208	2
P4144	SAMN05410904	PRJNA329301	MBKO00000000	2.191.601	219	123	2294	2055	60	3	53	4	179	2
P3478	SAMN03286964	PRJNA272784	JYIS00000000	2.150.567	343	103	2274	2063	59	5	53	1	152	1
Nm56	SAMN05437672	PRJNA329301	MCAC00000000	2.140.623	161	91	2223	2025	57	4	49	4	151	1
Nm287	SAMN05437673	PRJNA329301	MCAD00000000	2.125.606	98	98	2222	2004	55	3	48	4	163	1
Nm288	SAMN05437674	PRJNA329301	MCAE00000000	2.131.158	94	98	2238	2004	55	4	47	4	179	1
Nm292	SAMN05437675	PRJNA329301	MCAF00000000	2.112.711	45	105	2228	2019	54	3	47	4	155	1
Nm612	SAMN05437686	PRJNA329301	MCAG00000000	2.122.280	79	100	2223	2018	52	4	44	4	153	1
Nm638	SAMN05437771	PRJNA329301	MCAH00000000	2.110.969	52	99	2230	2002	55	3	48	4	173	1
Nm94	SAMN05437878	PRJNA329301	MCAI00000000	2.125.252	254	102	2221	1991	58	3	51	4	172	1
Nm322	SAMN05437879	PRJNA329301	MCAJ00000000	2.083.574	60	87	2201	1983	54	3	47	4	164	1
NmCRJ2	SAMN05437880	PRJNA329301	MCAK00000000	2.146.984	340	95	2236	2033	57	3	50	4	146	1

Table S2. Genomes from the Bacterial Isolate Genome Sequence platform (BIGSdb) hosted on the www.pubmlst.org/neisseria database used in this study.

ID	Isolate	Country	Year	Species	Serogroup	ST-MLST	Clonal complex MLST
1182	W-138a	Greece	1998	Neisseria meningitidis	A	103	ST-103 complex
1194	BM34	Greece	1996	Neisseria meningitidis	B	161	ST-103 complex
1197	BM34a	Greece	1996	Neisseria meningitidis	B	161	ST-103 complex
20248	M11 240100	UK	2011	Neisseria meningitidis	B	9239	ST-103 complex
21199	M11 240789	UK	2011	Neisseria meningitidis	B	103	ST-103 complex
26887	12028_2012	Ireland	2012	Neisseria meningitidis	B	1418	ST-103 complex
28060	M13 240448	UK	2013	Neisseria meningitidis	B	103	ST-103 complex
28067	M13 240458	UK	2013	Neisseria meningitidis	B	103	ST-103 complex
35259	09.2935.B	UK	2009	Neisseria meningitidis	B	103	ST-103 complex
35329	12.1092.Y	UK	2012	Neisseria meningitidis	B	103	ST-103 complex
35528	M13 240740	UK	2013	Neisseria meningitidis	B	8023	ST-103 complex
37832	M15 240053	UK	2015	Neisseria meningitidis	B	103	ST-103 complex
1038	8013	France	1989	Neisseria meningitidis	C	177	ST-18 complex
12672	53442	China	2004	Neisseria meningitidis	C	4821	ST-4821 complex
1592	0201/93	Czech Republic	1993	Neisseria meningitidis	C	103	ST-103 complex
19365	K1207	Italy	2007	Neisseria meningitidis	C	11	ST-11 complex/ET-37 complex
19505	S0108	Italy	2008	Neisseria meningitidis	C	11	ST-11 complex/ET-37 complex
20016	M10 240607	UK	2010	Neisseria meningitidis	C	5133	ST-103 complex
20079	M10 240705	UK	2010	Neisseria meningitidis	C	5133	ST-103 complex
20244	M11 240094	UK	2011	Neisseria meningitidis	C	5133	ST-103 complex
20397	M11 240347	UK	2011	Neisseria meningitidis	C	5133	ST-103 complex
20406	M11 240363	UK	2011	Neisseria meningitidis	C	5315	
21417	M12 240200	UK	2012	Neisseria meningitidis	C	5133	ST-103 complex
26733	LNP27256	France	2013	Neisseria meningitidis	C	11	ST-11 complex/ET-37 complex
26825	12026_2010	Ireland	2010	Neisseria meningitidis	C	1434	
28079	M12 240345	UK	2012	Neisseria meningitidis	C	5133	ST-103 complex
28096	M13 240201	UK	2013	Neisseria meningitidis	C	5315	
28101	M13 240398	UK	2013	Neisseria meningitidis	C	5133	ST-103 complex
29281	38277	South Africa	2012	Neisseria meningitidis	C	7180	ST-865 complex
30135	M05 240121	Unknown	NA	Neisseria meningitidis	C	344	
30196	M99 240595	UK	1999	Neisseria meningitidis	C	4095	
30223	C M-137	Unknown	NA	Neisseria meningitidis	C	344	
30279	2012 107	Portugal	2012	Neisseria meningitidis	C	839	ST-41/44 complex/Lineage 3
34548	9506	Brazil	2004	Neisseria meningitidis	C	33	ST-32 complex/ET-5 complex
34550	12888	Brazil	2004	Neisseria meningitidis	C	639	ST-32 complex/ET-5 complex
34570	73696	Brazil	1973	Neisseria meningitidis	C	8813	
34571	73704	Brazil	1973	Neisseria meningitidis	C	11	ST-11 complex/ET-37 complex
34576	81858	Vietnam	1981	Neisseria meningitidis	C	344	
34601	2002020	Cameroon	2001	Neisseria meningitidis	C	32	ST-32 complex/ET-5 complex
34613	M13255	USA	2005	Neisseria meningitidis	C	32	ST-32 complex/ET-5 complex
34614	M13265	USA	2004	Neisseria meningitidis	C	32	ST-32 complex/ET-5 complex
34619	NM126	USA	1996	Neisseria meningitidis	C	11	ST-11 complex/ET-37 complex
34620	NM133	USA	1996	Neisseria meningitidis	C	11	ST-11 complex/ET-37 complex
34624	NM1482	USA	2003	Neisseria meningitidis	C	1287	ST-11 complex/ET-37 complex
34625	NM1495	USA	2003	Neisseria meningitidis	C	11	ST-11 complex/ET-37 complex
34629	NM23	USA	1992	Neisseria meningitidis	C	11	ST-11 complex/ET-37 complex
34640	NM313	USA	2003	Neisseria meningitidis	C	11	ST-11 complex/ET-37 complex

34648	NM32	USA	1993	Neisseria meningitidis	C	11	ST-11 complex/ET-37 complex
34652	NM35	USA	1993	Neisseria meningitidis	C	11	ST-11 complex/ET-37 complex
34653	NM36	USA	1993	Neisseria meningitidis	C	11	ST-11 complex/ET-37 complex
34658	NM43	USA	1993	Neisseria meningitidis	C	11	ST-11 complex/ET-37 complex
34667	NM82	USA	1995	Neisseria meningitidis	C	11	ST-11 complex/ET-37 complex
34669	NM94	USA	1995	Neisseria meningitidis	C	11	ST-11 complex/ET-37 complex
34670	NM95	USA	1995	Neisseria meningitidis	C		
35366	12.4885.X	UK	2012	Neisseria meningitidis	C	6610	
35367	12.4886.L	UK	2012	Neisseria meningitidis	C	6610	
35551	M14 240020	UK	2014	Neisseria meningitidis	C	5133	ST-103 complex
36446	2419	Italy	2012	Neisseria meningitidis	C	11	ST-11 complex/ET-37 complex
698	FAM18	USA	1983	Neisseria meningitidis	C	11	ST-11 complex/ET-37 complex
21150	M11 240601	Malta	2011	Neisseria meningitidis	NG	103	ST-103 complex
960	0048/93	Czech Republic	1993	Neisseria meningitidis	NG	103	ST-103 complex
21505	M12 240332	UK	2012	Neisseria meningitidis	Y	5987	ST-103 complex
28176	M12 240748	UK	2012	Neisseria meningitidis	Y	103	ST-103 complex
34622	NM140	USA	1996	Neisseria meningitidis	Z	2981	ST-103 complex
34628	NM183	USA	1998	Neisseria meningitidis	Z	5467	ST-103 complex
34634	NM2781	USA	1998	Neisseria meningitidis	Z	6937	ST-103 complex
34662	NM576	USA	2003	Neisseria meningitidis	Z	5467	ST-103 complex
35289	10.2631.L	UK	2010	Neisseria meningitidis	Z	103	ST-103 complex
35957	890326	The Netherlands	1989	Neisseria meningitidis	Z	28	ST-103 complex
985	0084/93	Czech Republic	1993	Neisseria meningitidis	Z	1002	ST-103 complex
26322	R49	UK	2011	Neisseria meningitidis		103	ST-103 complex

Table S3. Toxin-Antitoxin (TA) systems found in FAM18 and Nm56 genomes. The TA prediction was performed by TAFinder web-based tool (http://202.120.12.133/TAfinder). The Nm56 genome harbor six TA systems, two of them formed by the *yeb-yecA* and *prlF-yhaV* genes localized in the genomic island ICE*Nm*CC103.

TA	Gene	Family	FAM18	Nm56	ICENm56
1	NMC0198	-	1	1	0
	NMC0197	-	1	1	0
2	NMC0332	*relE*-like	1	1	0
	NMC0331	*Xre*-like	1	1	0
3	NMC0879	*MazF*-like	1	1	0
	NMC0880	*AbrB*-like	1	1	0
4	NMC2019	*MazF*-like	1	1	0
	NMC2018	*AbrB*-like	1	1	0
5	NMC1584	*PIN*-like	1	0	0
	NMC1585	*PHD*-like	1	0	0
6	*yeb*	*GNAT*-like	0	1	1
	yecA	*RHH*-like	0	1	1
7	*prlF*	*relE*-like	0	1	1
	yhaV	*AbrB*-like	0	1	1

1: Presence
0: Absence

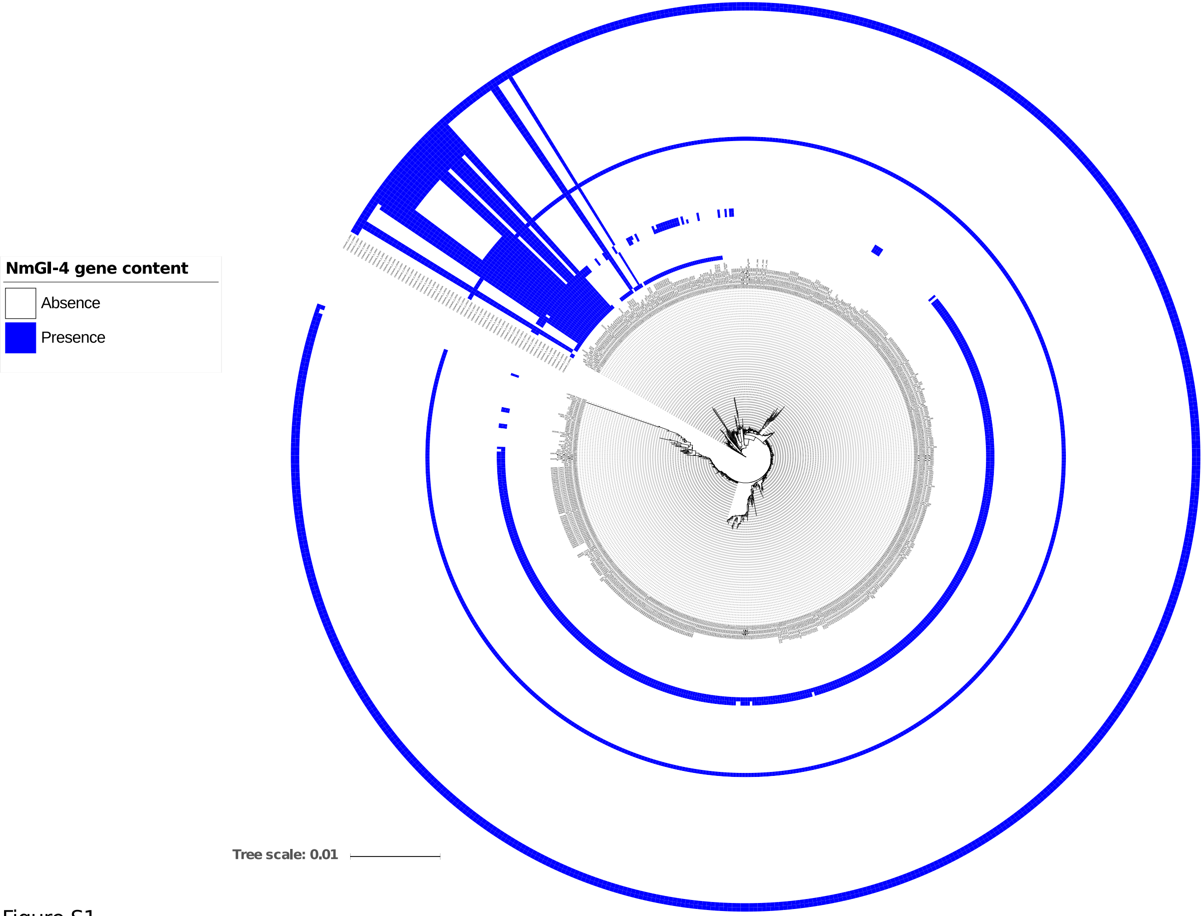

Figure S1

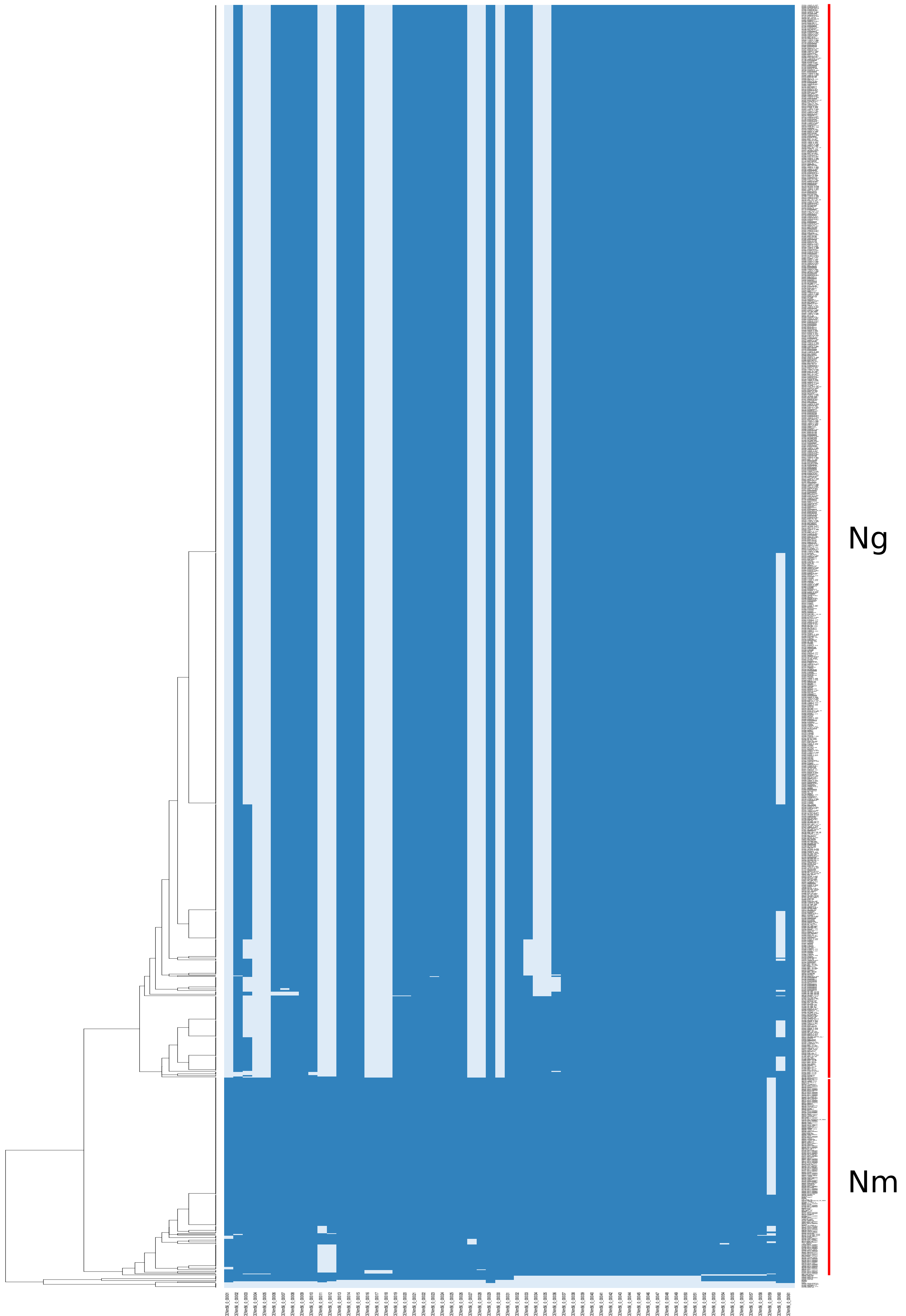

Figure S2

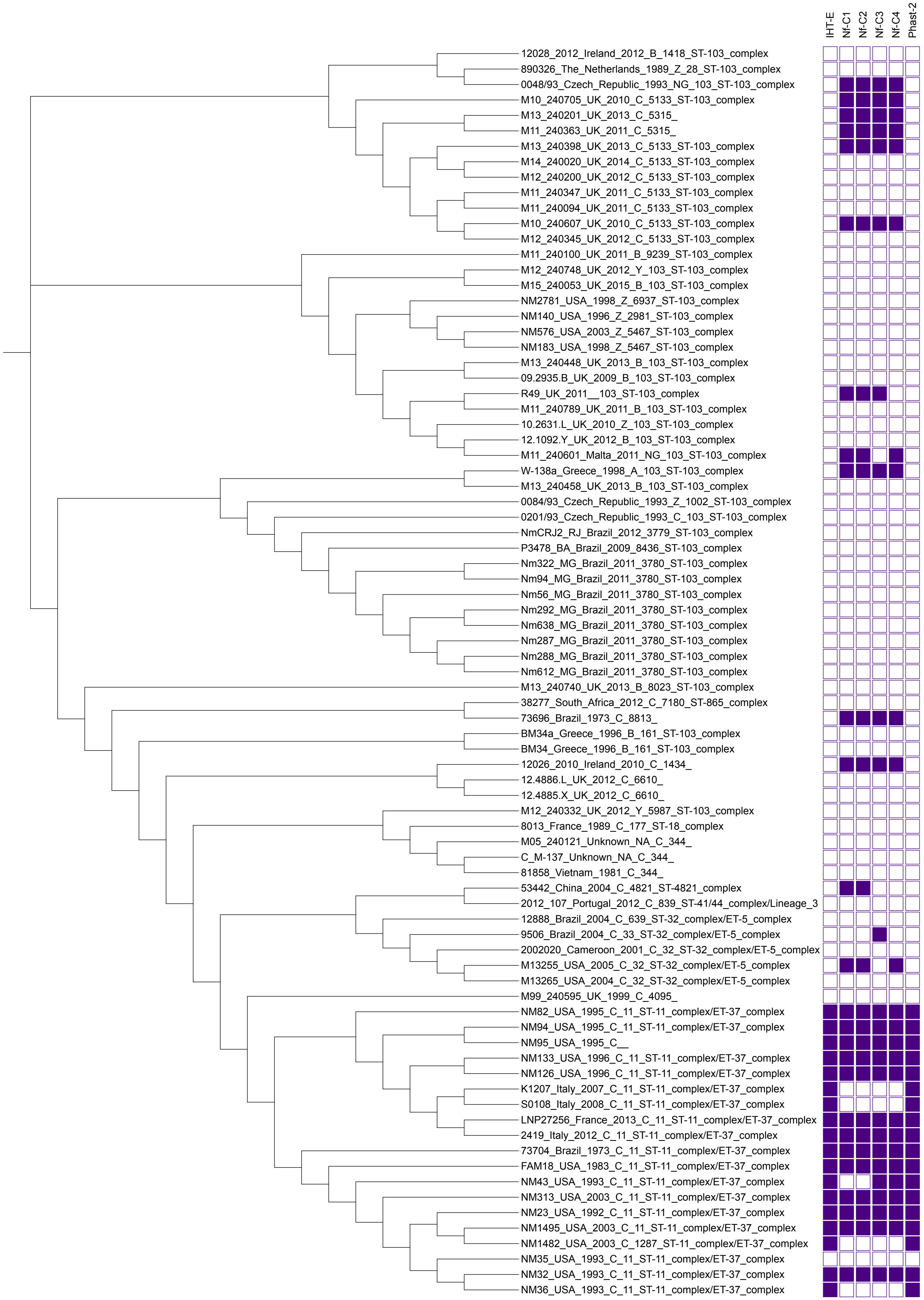
IHT-E
Nf-C1
Nf-C2
Nf-C3
Nf-C4
Phast-2
12028_2012_Ireland_2012_B_1418_ST-103_complex
890326_The_Netherlands_1989_Z_28_ST-103_complex
0048/93_Czech_Republic_1993_NG_103_ST-103_complex
M10_240705_UK_2010_C_5133_ST-103_complex
M13_240201_UK_2013_C_5315_
M11_240363_UK_2011_C_5315_
M13_240398_UK_2013_C_5133_ST-103_complex
M14_240020_UK_2014_C_5133_ST-103_complex
M12_240200_UK_2012_C_5133_ST-103_complex
M11_240347_UK_2011_C_5133_ST-103_complex
M11_240094_UK_2011_C_5133_ST-103_complex
M10_240607_UK_2010_C_5133_ST-103_complex
M12_240345_UK_2012_C_5133_ST-103_complex
M11_240100_UK_2011_B_9239_ST-103_complex
M12_240748_UK_2012_Y_103_ST-103_complex
M15_240053_UK_2015_B_103_ST-103_complex
NM2781_USA_1998_Z_6937_ST-103_complex
NM140_USA_1996_Z_2981_ST-103_complex
NM576_USA_2003_Z_5467_ST-103_complex
NM183_USA_1998_Z_5467_ST-103_complex
M13_240448_UK_2013_B_103_ST-103_complex
09.2935.B_UK_2009_B_103_ST-103_complex
R49_UK_2011__103_ST-103_complex
M11_240789_UK_2011_B_103_ST-103_complex
10.2631.L_UK_2010_Z_103_ST-103_complex
12.1092.Y_UK_2012_B_103_ST-103_complex
M11_240601_Malta_2011_NG_103_ST-103_complex
W-138a_Greece_1998_A_103_ST-103_complex
M13_240458_UK_2013_B_103_ST-103_complex
0084/93_Czech_Republic_1993_Z_1002_ST-103_complex
0201/93_Czech_Republic_1993_C_103_ST-103_complex
NmCRJ2_RJ_Brazil_2012_3779_ST-103_complex
P3478_BA_Brazil_2009_8436_ST-103_complex
Nm322_MG_Brazil_2011_3780_ST-103_complex
Nm94_MG_Brazil_2011_3780_ST-103_complex
Nm56_MG_Brazil_2011_3780_ST-103_complex
Nm292_MG_Brazil_2011_3780_ST-103_complex
Nm638_MG_Brazil_2011_3780_ST-103_complex
Nm287_MG_Brazil_2011_3780_ST-103_complex
Nm288_MG_Brazil_2011_3780_ST-103_complex
Nm612_MG_Brazil_2011_3780_ST-103_complex
M13_240740_UK_2013_B_8023_ST-103_complex
38277_South_Africa_2012_C_7180_ST-865_complex
73696_Brazil_1973_C_8813_
BM34a_Greece_1996_B_161_ST-103_complex
BM34_Greece_1996_B_161_ST-103_complex
12026_2010_Ireland_2010_C_1434_
12.4886.L_UK_2012_C_6610_
12.4885.X_UK_2012_C_6610_
M12_240332_UK_2012_Y_5987_ST-103_complex
8013_France_1989_C_177_ST-18_complex
M05_240121_Unknown_NA_C_344_
C_M-137_Unknown_NA_C_344_
81858_Vietnam_1981_C_344_
53442_China_2004_C_4821_ST-4821_complex
2012_107_Portugal_2012_C_839_ST-41/44_complex/Lineage_3
12888_Brazil_2004_C_639_ST-32_complex/ET-5_complex
9506_Brazil_2004_C_33_ST-32_complex/ET-5_complex
2002020_Cameroon_2001_C_32_ST-32_complex/ET-5_complex
M13255_USA_2005_C_32_ST-32_complex/ET-5_complex
M13265_USA_2004_C_32_ST-32_complex/ET-5_complex
M99_240595_UK_1999_C_4095_
NM82_USA_1995_C_11_ST-11_complex/ET-37_complex
NM94_USA_1995_C_11_ST-11_complex/ET-37_complex
NM95_USA_1995_C__
NM133_USA_1996_C_11_ST-11_complex/ET-37_complex
NM126_USA_1996_C_11_ST-11_complex/ET-37_complex
K1207_Italy_2007_C_11_ST-11_complex/ET-37_complex
S0108_Italy_2008_C_11_ST-11_complex/ET-37_complex
LNP27256_France_2013_C_11_ST-11_complex/ET-37_complex
2419_Italy_2012_C_11_ST-11_complex/ET-37_complex
73704_Brazil_1973_C_11_ST-11_complex/ET-37_complex
FAM18_USA_1983_C_11_ST-11_complex/ET-37_complex
NM43_USA_1993_C_11_ST-11_complex/ET-37_complex
NM313_USA_2003_C_11_ST-11_complex/ET-37_complex
NM23_USA_1992_C_11_ST-11_complex/ET-37_complex
NM1495_USA_2003_C_11_ST-11_complex/ET-37_complex
NM1482_USA_2003_C_1287_ST-11_complex/ET-37_complex
NM35_USA_1993_C_11_ST-11_complex/ET-37_complex
NM32_USA_1993_C_11_ST-11_complex/ET-37_complex
NM36_USA_1993_C_11_ST-11_complex/ET-37_complex

Figure S3

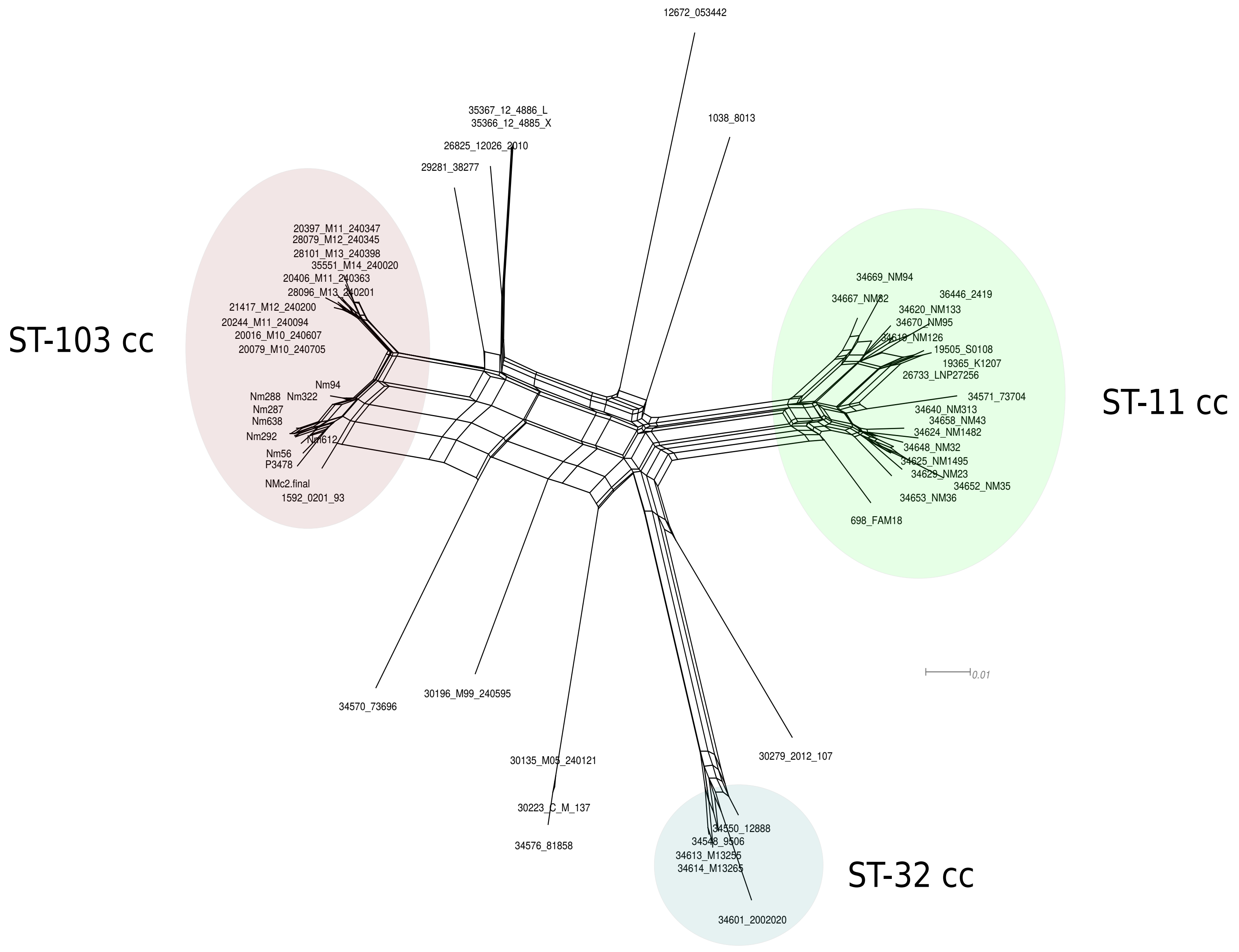

12672_053442
1038_8013
35367_12_4886_L
35366_12_4885_X
26825_12026_2010
29281_38277
ST-103 cc
20397_M11_240347
28079_M12_240345
28101_M13_240398
35551_M14_240020
20406_M11_240363
28096_M13_240201
21417_M12_240200
20244_M11_240094
20016_M10_240607
20079_M10_240705
Nm94
Nm288
Nm322
Nm287
Nm638
Nm292
Nm612
Nm56
P3478
NMc2.final
1592_0201_93
ST-11 cc
34669_NM94
34667_NM82
36446_2419
34620_NM133
34670_NM95
34619_NM126
19505_S0108
19365_K1207
26733_LNP27256
34571_73704
34640_NM313
34658_NM43
34624_NM1482
34648_NM32
34625_NM1495
34629_NM23
34652_NM35
34653_NM36
698_FAM18
0.01
34570_73696
30196_M99_240595
30135_M05_240121
30223_C_M_137
34576_81858
30279_2012_107
ST-32 cc
34550_12888
34548_9506
34613_M13255
34614_M13265
34601_2002020

Figure S4
